# MEMS-Based Handheld Fourier Domain Doppler Optical Coherence Tomography for Intraoperative Microvascular Anastomosis Imaging

**DOI:** 10.1371/journal.pone.0114215

**Published:** 2014-12-04

**Authors:** Yong Huang, Georg J. Furtmüller, Dedi Tong, Shan Zhu, W. P. Andrew Lee, Gerald Brandacher, Jin U. Kang

**Affiliations:** 1 Department of Electrical and Computer Engineering, Johns Hopkins University, 3400 N. Charles Street, Baltimore, Maryland, 21218, United States of America; 2 Department of Plastic and Reconstructive Surgery, Vascularized Composite Allotransplantation (VCA) Laboratory, Johns Hopkins University School of Medicine, 720 Rutland Avenue, Ross 749D, Baltimore, Maryland, 21205, United States of America; 3 Department of Hand Surgery, Beijing Jishuitan Hospital, 31 Xinjiekou East Street, Xicheng District, Beijing, 10035, China; 4 Peking Union Medical College and Chinese Academy of Medical Sciences, Department of Plastic and Reconstructive Surgery, Plastic Surgery Hospital, 3 Ba-Da-Chu Road, Shijingshan District, Beijing, 10044, China; University of Manchester, United Kingdom

## Abstract

**Purpose:**

To demonstrate the feasibility of a miniature handheld optical coherence tomography (OCT) imager for real time intraoperative vascular patency evaluation in the setting of super-microsurgical vessel anastomosis.

**Methods:**

A novel handheld imager Fourier domain Doppler optical coherence tomography based on a 1.3-µm central wavelength swept source for extravascular imaging was developed. The imager was minimized through the adoption of a 2.4-mm diameter microelectromechanical systems (MEMS) scanning mirror, additionally a 12.7-mm diameter lens system was designed and combined with the MEMS mirror to achieve a small form factor that optimize functionality as a handheld extravascular OCT imager. To evaluate *in-vivo* applicability, super-microsurgical vessel anastomosis was performed in a mouse femoral vessel cut and repair model employing conventional interrupted suture technique as well as a novel non-suture cuff technique. Vascular anastomosis patency after clinically successful repair was evaluated using the novel handheld OCT imager.

**Results:**

With an adjustable lateral image field of view up to 1.5 mm by 1.5 mm, high-resolution simultaneous structural and flow imaging of the blood vessels were successfully acquired for BALB/C mouse after orthotopic hind limb transplantation using a non-suture cuff technique and BALB/C mouse after femoral artery anastomosis using a suture technique. We experimentally quantify the axial and lateral resolution of the OCT to be 12.6 µm in air and 17.5 µm respectively. The OCT has a sensitivity of 84 dB and sensitivity roll-off of 5.7 dB/mm over an imaging range of 5 mm. Imaging with a frame rate of 36 Hz for an image size of 1000(lateral)×512(axial) pixels using a 50,000 A-lines per second swept source was achieved. Quantitative vessel lumen patency, lumen narrowing and thrombosis analysis were performed based on acquired structure and Doppler images.

**Conclusions:**

A miniature handheld OCT imager that can be used for intraoperative evaluation of microvascular anastomosis was successfully demonstrated.

## Introduction

Vascular anastomosis–the surgical connection of two blood vessels–is a common procedure used in various surgical subspecialties. In particular, it is a critical constituent of reconstructive microsurgery, vascular surgery, and transplant surgery. The conventional suture-based anastomosis technique has seen many technological advances since the introduction of the triangulation method by Carrel [1]. The advances range from improved surgical microscopes to improved suture material and vascular coupling devices [2], thermo-reversible poloxamers [3], and non-suture cuff techniques [4]. However, quality assessment of surgical outcomes depends merely on the experience of the surgeon and involves visual inspection of the suture site as well as all tissues involved and perfused by the vascular pedicle. Thus, despite the overall progress pushing the limits of microsurgical possibilities, no significant clinically relevant advances have been made allowing for the standardized postoperative evaluation of microvascular anastomosis patency. In the field of imaging, technologies such as near-infrared laser angiography may provide surrogate information about perfusion and drainage by measuring the accumulation of fluorescent dyes, however an application providing real time surgical guidance and the opportunity of immediate postoperative microvascular anastomosis evaluation is deemed to be the ultimate goal. In the era of super-microsurgery, there is a critical need for devices that can evaluate the surgical outcome objectively and not solely based on a surgeons’ subjective evaluation and accumulated experience over the years. Such devices can also aid in training and evaluation of trainees.

Optical coherence tomography (OCT) has been successfully used in pre-clinical and clinical applications due to its high resolution simultaneous structure and Doppler flow imaging [5]. OCT has been demonstrated with the advantages of fast temporal and spatial resolution compared to other medical imaging modalities such as MRI, CT and ultrasound tomography. Our lab recently demonstrated the capability of a real-time graphics processing units accelerated Phase resolved Doppler OCT (PRDOCT) system for suturing guidance and postoperative evaluation of microvascular anastomosis [6], [7]. Compared to laser speckle contrast blood flow imaging, PRDOCT has the advantage of simultaneous high resolution depth-resolved structure and blood flow information [8]. While Doppler optical microangiography imaging (DOMAG) can provide better performance than PRDOCT, it requires more complicated system hardware configuration [9]. Intraoperative application of OCT requires a task-oriented OCT system optomechanical design to achieve bench-top to bed-side clinical translation. Different approaches have been explored by researchers such as integration of OCT to surgical microscope for vitreoretinal surgery [10] and miniature handheld probes via fiber tip resonant vibration [5], [11], scanning mirrors combined with GRIN needles [12], endoscopic MEMS probe [13], and MEMS mirrors-based handheld imaging instrument [14], [15]. 2D MEMS mirrors that scan in two axes as an alternative to the larger galvanometer scanner have been used extensively to generate 3D volume images due to their low-cost, compact size, and fast imaging speed [15]. MEMS mirror scanning is conventional linear raster-scanning, which is uniform in spatial sampling and the acquired image is more intuitive to understand compared to fiber tip resonant vibration in 3D volume image generation mode. Due to the easy access of vessels during the open anastomosis surgery, we chose to develop a handheld imager using a 2D MEMS mirror-based scanner. The ergonomic design aim was that surgeons can use the imager as a typical handheld surgical tool. By making the OCT system portable and the probe miniature, the structure and Doppler OCT imaging can be readily available for surgeons during the procedure.

In this work, we evaluated handheld OCT imager by performing the intraoperative evaluation of microvascular anastomosis using a mouse model. The proposed handheld MEMS scanning mirror-based imager is compact, light-weight, and easy to manipulate. It can provide real-time simultaneous structure and Doppler flow imaging at a speed of 36 frames per second while each frame consists of 1000×512 (lateral×axial) pixels. The Doppler flow imaging range was characterized to be from 0.363 mm/s to 16.3 mm/s in both directions parallel to the scanning beam.

## Materials and Methods

### Ethnic Statement

Johns Hopkins University Animal Care and Use Committee approved all experiments with the approval number of MO12M348 and the animals were handled in accordance with the Johns Hopkins University Animal Care and Use Committee guidelines. Animals were housed under standard rodent housing conditions (24C, 12∶12 h dark/light cycle), food and water was provided ad libitum. Analgesia with Buprenorphine (Dose: 0.05 mg/kg) was administered 1 hour prior to surgery and then at an interval of 60 min thereafter. Animals were operated under sterile conditions and put on a heating pad to maintain body temperature and all efforts were made to minimize suffering. Non-invasive imaging procedures were performed immediately after the surgical procedure was completed. At the end of the experiments animals were sedated with an overdose of isoflurane inhalation anesthesia and euthanized by cervical dislocation.

### Animals

Male 6–8 week old BALB/C mice with a BW of 33±2.8 g (n = 2) were obtained from Jackson Laboratories. Animals were anesthetized using oxygen and isoflurane mixed gas and placed on a heating pad to maintain the body temperature. In Mouse 1, femoral artery end-to-end anastomoses was performed with a conventional suture technique. Mouse 2 received an orthotopic hind limb transplantation surgery with a non-suture cuff-based technique for revascularization. Both femoral vein and artery were anastomosed from the donor limb to the recipient animal’s vessels.

### Microvascular Anastomosis Procedures

**Figure 1:**
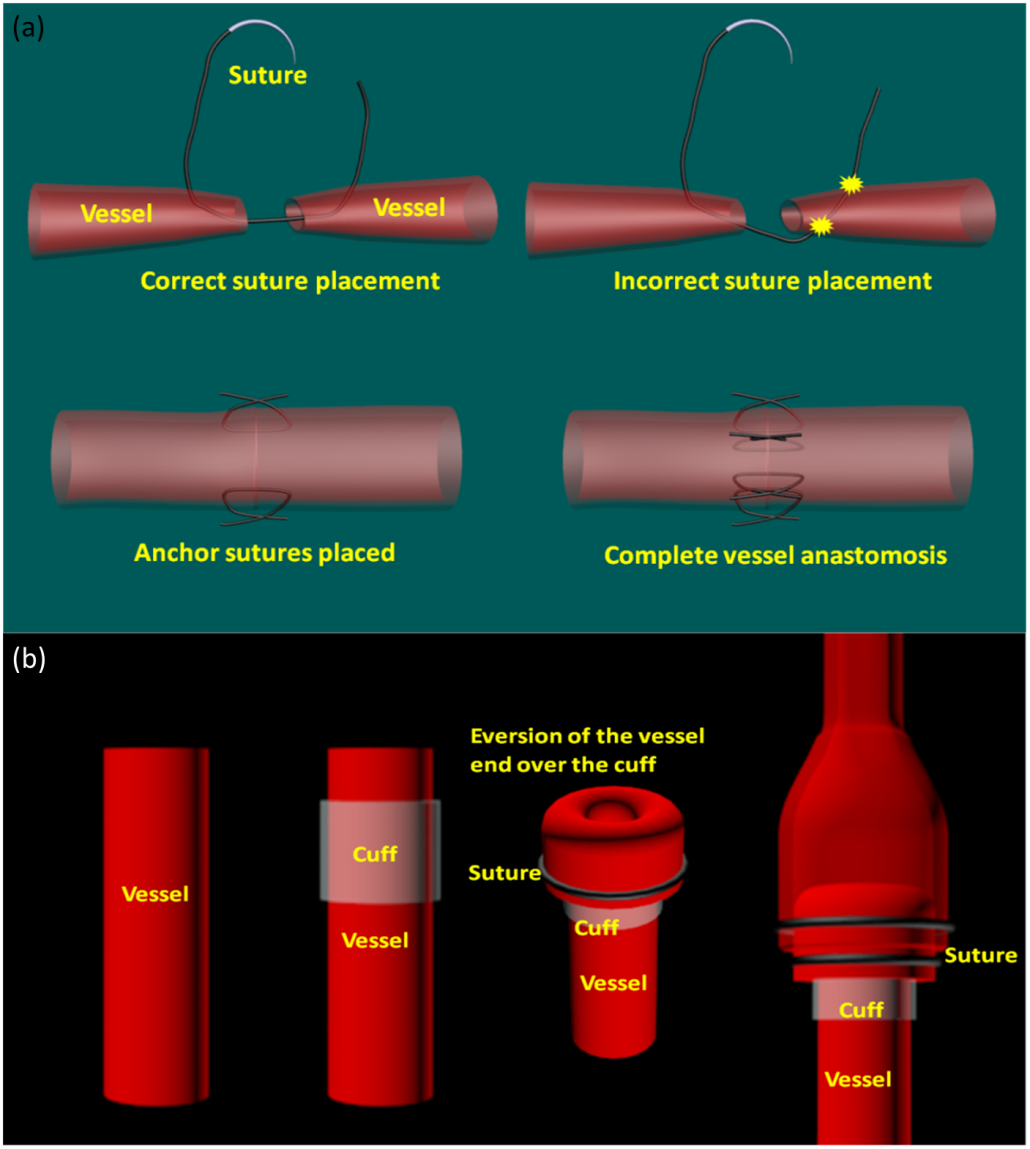
Illustration of microvascular anastomosis procedures. (a) suture based vascular anastomosis; (b) cuff based non-suture technique. Cuff is mounted over the vessel end and the end is everted over the cuff. The other cut end of the vessel is then pulled over and tied to create intima-to-intima contact.

Conventional interrupted suture technique: Supermicrovascular anastomosis was performed in a mouse femoral artery cut and repair model using interrupted suture technique (11-0 Nylon, S&T, Neuhausen, Switzerland). Femoral vessels were dissected, cut and the proximal and distal stump mounted on an approximating clamp (S&T, Neuhausen, Switzerland) to prevent blood flow and provide optimal surgical exposure. Fig. 1(a) illustrates the procedures of suture based vascular anastomosis. Sutures are placed in the collapsed vessel across the full thickness of the vessel wall (adventitia, media and intima) on one end and then from intima to adventitia in the other vessel end. Appropriate approximation of proximal and distal vessel stumps was achieved with 6–8 sutures.

Non-suture cuff technique: Our group pioneered the non-suture-cuff technique, which provides a rapid and reliable method for performing sub-millimeter anastomosis [4], [16]. Fig. 1(b) shows the procedures of the sutureless cuff technique. First, a biocompatible cuff with proper diameter to fit the size of vessel is chosen. The cuff is mounted on the distal vessel end, which is subsequently everted and secured over the cuff using 10-0 circular ties. The distal vessel end with the cuff is now secured in an approximating clamp to provide optimal stability and surgical exposure. The proximal vessel is pulled over the vessel-cuff complex providing intima-to-intima contact. Release of the approximating clamp reestablished blood flow and patency could be evaluated macroscopically.

Conventional rubber material as a background, is placed to both facilitate the microsurgical procedure and provide optimal separation from surrounding tissue for optimal and clear imaging conditions.

### Handheld-Probe Design

**Figure 2:**
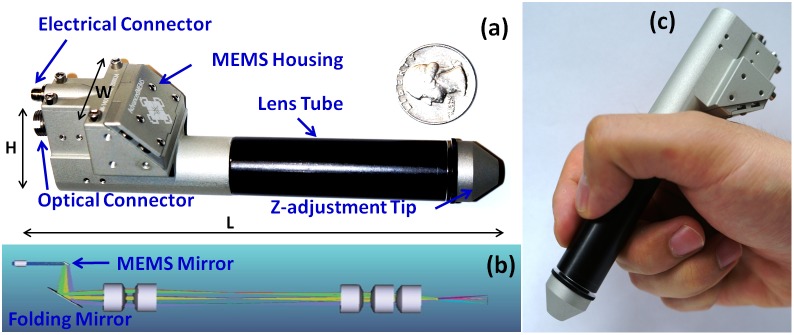
Handheld probe illustration. (a) Probe compared to a quarter coin (H: height 37.24 mm, W: width 32.43 mm, L: length 136.7 mm). (b) Optics layout inside the probe. (c) Image of the probe held by hand.

Fig. 2(a) illustrated the optomechanical design of the probe. It consists of mainly three parts: gray MEMS housing part with optical and electrical connectors integrated to it, black lens tube part and a gray axial distance adjustment tip. To achieve light weight, the probe is made of aluminum and the weight of the probe is measured to be 88 g. An FC/APC collimator with an output beam diameter of 1.5 mm was used as the optical input of the probe. MEMS scanning mirror (AdvancedMEMS Inc.) with a diameter of 2.4 mm was chosen to accommodate and stir the beam to perform scanning. MEMS scanning waveforms were supplied through the electrical input connector located besides the optical input. A folding mirror was used to fold the beam to a five half inch lens system, which is shown in Fig. 2(b). Four half inches achromatic doublets (Thorlabs, AC127-050-C) form a quasi-telecentric setup with two pairs of identical achromatic lenses to minimize spherical aberration. One half inch achromatic lens with focal length of 30 mm (Thorlabs, AC127-030-C) is used as the objective lens to focus the imaging beam. The first pair of two lenses was placed within the gray MEMS housing part and the remaining three lenses were placed in the lens tube. The length of the probe is 136.7 mm with 17.2 mm axial distance adjustment range. The axial distance adjustment tip was designed for fine-tuning the sample position relative to the zero-delay reference. It also gives us some flexibility when later longer focal length lens is used. The height of the probe is 37 mm and width is 32 mm at the MEMS housing and collimator region; after that the probe is a tube structure with an outer diameter of 23 mm. A photograph showing a normal hand holding the probe is shown in Fig. 2(c).

### Swept Source-OCT System

**Figure 3:**
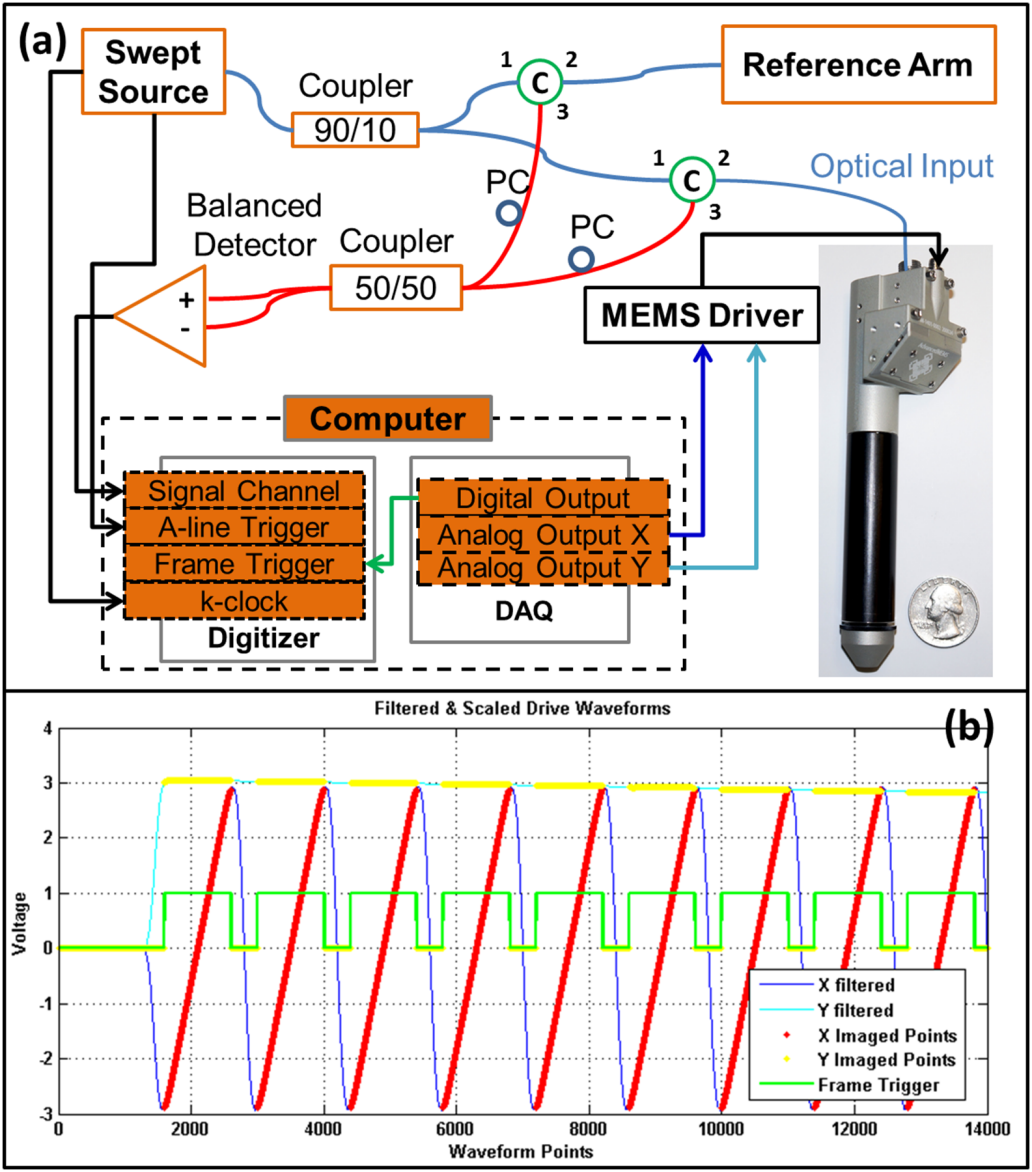
Schematic system setup and MEMS mirror control waveforms. (a) system configuration (C: circulator, PC: polarization controller, DAQ: data acquisition card.) (b) filtered and scaled waveforms generated by DAQ to MEMS driver to control the scanning mirror.

Fig. 3(a) presents the system configuration. The swept source laser (Axsun Technologies, Inc.) with a central wavelength of 1310 nm, tuning range of 100 nm (from 1260 nm to 1360 nm), and A-scan rate of 50 kHz was used. Balanced detector (Thorlabs, PDB480C AC) is utilized to detect the interference signal to remove the common DC background. We used a quad-core@3.0 GHz Dell Precision T7600 workstation to host a digitizer (AlazarTech, ATS9350), a DAQ card (National Instrument, PCI-6221), one graphics processing unit (GPU) (NVIDIA, Geforce GTX580). A-line trigger and k-clock signal from the laser source are routed to the specific ports of the digitizer for equal k-space A-line data sampling. The DAQ card generates MEMS control waveforms and synchronized digital output as frame trigger to the digitizer for the acquisition start of a frame. GPU is used to achieve real-time signal processing and image display. All the scanning control and synchronization, data acquisition, image processing, and rendering were performed on a multi-thread, CPU-GPU heterogeneous computing system. The hybrid computing system and a customized user interface was designed and programmed through Qt 4.8.5 and C++ (Microsoft Visual Studio, 2008). We used computer unified device architecture (CUDA) version 5.0 from NVIDIA to program the GPU for general purpose computations.

Fig. 3(b) shows the carefully designed control waveforms generated by the DAQ to the MEMS driver and digitizer for C-mode raster scanning imaging. An internal clock with a sampling rate of 50 kHz was used as the clock source for the fast-moving X and slow-moving Y waveforms. Each waveform consisted of 252 sections while the first and the last section were used to put and return the MEMS mirror at a global starting point in the Y direction. Each section consists of 1400 sampling points. Fast-moving X waveform is quasi-sinusoidal with the first and last 200 points of the section serving as the lead-in and lead-out points to put the mirror from the zero point to acquisition start point and guide the mirror back from the acquisition end point to the zero point in X direction. A synchronized digital output from the DAQ with a rising edge as a frame trigger was routed to the digitizer frame start trigger input, shown as green lines in Fig. 3(b). Once the trigger is detected, the system acquires 1000 A-lines in the linear region, which is shown as the red dots for X imaged points and yellow dots for Y imaged points. Each A-line consists of 1024 sampling points in the spectral domain. The waveforms are updated periodically and iteratively through these 252 sections once a section is generated. For B-mode imaging, we simply set the Y output to be zero all the time. Scan patterns can be switched at any time with a delay of a few tens of milliseconds from B-mode to C-mode. Since the signal generation source clock is 50 kHz and each section consists of 1400 points, this gives us an effective imaging speed of 36 frames per second. It needs to be pointed out that the imaging frame rate can be further increased by reducing the designed imaged points or reducing the linearity of the scanning region by allowing the MEMS mirror to perform more resonant scanning.

### Imaging Protocol and Data Quantification Analysis

The OCT system was running at 36 fps with each frame size of 1000×512 pixels, corresponds to 1.5 mm (fast X)×5 mm (axial) at 2D scanning mode. The 3D volume scanning mode consists of 252 frames in the slow Y axis, which took around 7 seconds to complete. Thus 252×1000×512 (1.5×1.5×5 mm^3^) voxels is acquired to cover the area of interest. Postoperative manual segmentation and image analysis was through ImageJ 1.46r (NIH). Inner lumen area, lumen patency rate, lumen narrowing rate and thrombosis percentage were extracted.

## Results and Discussion

**Figure 4:**
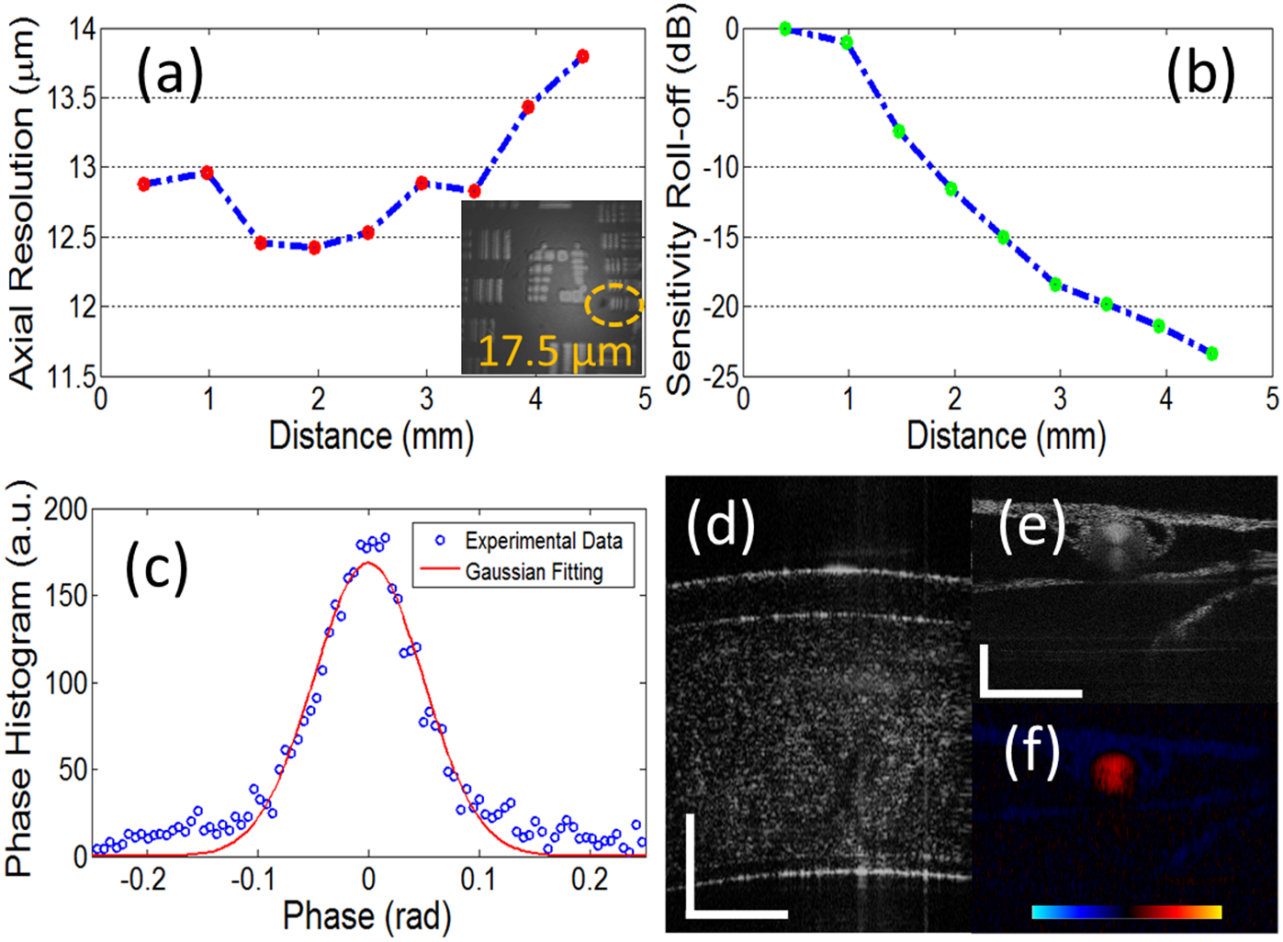
OCT System performance evaluation. (a) measured axial resolution over the imaging range, inset: lateral resolution image of USAF target; (b) sensitivity roll-off over the imaging range; (c) measured phase stability of a stationary mirror. (d) 5 frames averaged cornea imaging. (e) 5 frames averaged chicken embryo imaging. (f) 5 frames averaged corresponding Doppler flow imaging of (e) (scale bar: 500 **µ**m, Doppler color bar range: **−**16.3 mm/s to 16.3 mm/s).

After assembling the probe and setting up the system, we carefully evaluated the probe performance experimentally. Prior to the evaluation we carefully matched the dispersion of the sample and reference through hardware first. Residual second-order dispersion matching was achieved through our real-time numerical dispersion compensation method [12]. We first analyzed axial resolution of the system. Fig. 4(a) shows the measured results over the imaging range. An average axial resolution of 12.6 µmis achieved. We evaluated the lateral resolution of our system by imaging a USAF-1951 resolution target; the result is shown in Fig. 4(a) inset. We can resolve up to the Group 5 Element 6, which implies a lateral resolution of 17.5 µm. This is very close to the theoretically predicted value of 16 µmusing the formula 0.61fλ/D assuming a focal length of objective lens (f) 30 mm and beam diameter (D) of 1.5 mm, where λ is the central wavelength of light source (1.31 µm). The system sensitivity was measured to be 84 dB by putting a mirror as the sample at 0.4 mm from the zero-delay line and a neutral density filter in front of the mirror. The system sensitivity roll-off is shown in Fig. 4(b); the average decay is 5.7 dB/mm. We compared these values to a standard galvanometer-based scanning system using an LSM04 3X OCT scan lens, which has a sensitivity of 98 dB and a 4.7 dB/mm sensitivity roll-off. Reduced sensitivity is probably due to the increased signal loss in the current MEMS probe compared to a scan lens-based system. Larger sensitivity decay is due to the reduced depth of focus of the current lens that has a focal length of 30 mm compared to 54 mm of LSM04. Therefore further optical system optimization of the probe through customizing lens design and coating will be achieved.

To evaluate the phase stability and thus the Doppler imaging sensitivity of the imager, we imaged a stationary mirror and extracted the phase value of the mirror surface position. A phase histogram is plotted in Fig. 4(c). By Gaussian fitting the experimental data, the standard deviation of the fitting curve gave us a standard deviation of 70 mrad, which is due to the combination of the system noise, MEMS mirror-scanning noise, and the environmental noise. The Doppler flow imaging speed range thus is calculated to be from 0.363 mm/s to 16.3 mm/s in both directions parallel to the scanning beam using v(axical) = λΔφ/4πΔt), where Δt equals 20 µs.

To evaluate the structure and Doppler imaging capability of our system before animal experiments, we imaged an *ex-vivo* cow eye cornea shown in Fig. 4(d); all layers are clearly visualized. We also imaged an *in-vivo* chorioallantoic membrane (CAM) of a 16-day-aged chick embryo. Fig. 4(e) shows the structure image of one vessel cross-section. Fig. 4(f) shows the corresponding Doppler image. We can see the nearly parabolic distribution of the blood flow within the vessel lumen.

**Figure 5:**
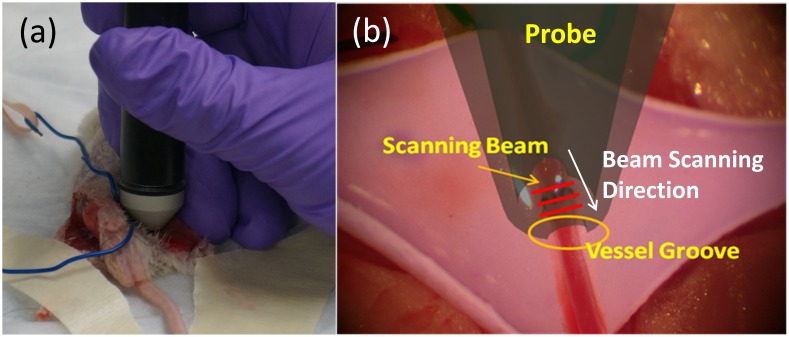
Application of handheld probe in the mouse experiment. (a) Handheld probe investigating the surgical site of an experimented mouse; (b) Illustration of how the beam scans across the vessel region (red lines indicate that fast B-mode scanning beam, white arrow indicates slow beam scanning direction, yellow circles marks vessel groove to be implemented in our future probe optimization).

**Figure 6:**
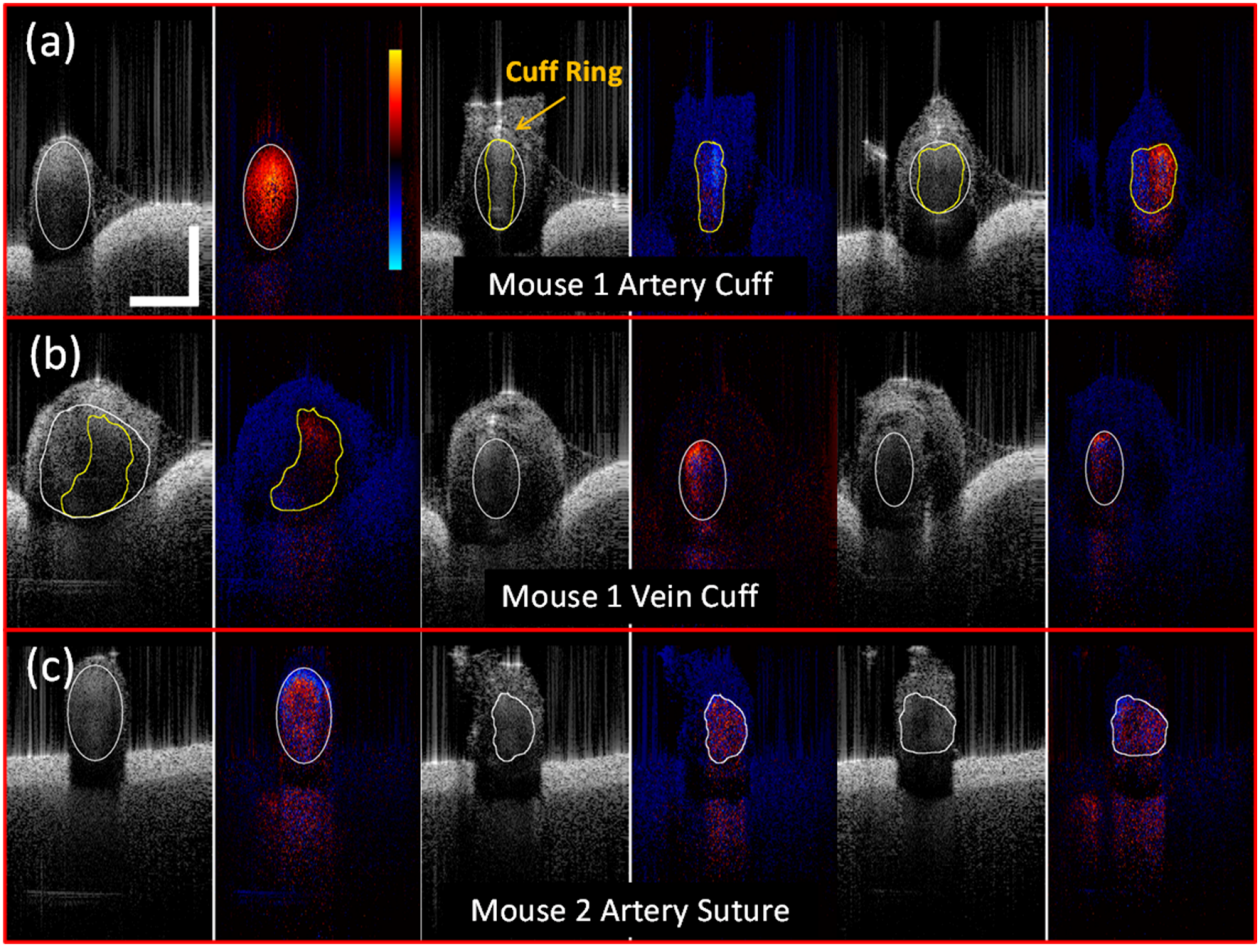
Selective frames of simultaneous structure and Doppler imaging of the surgical sites from distal (left) to proximal (right). White lines represent manually segmented blood vessel inner lumen, red lines represent manually segmented thrombosis, blue lines outline the blood flowing area within the vessel inner lumen. (a) Mouse 1 artery (Video S1); (b) Mouse 1 vein (Video S2) and (c) Mouse 2 artery (Video S3). Videos were played back at 24 frames per second. Images were cropped to best fit the area of interest (scale bar: 500 µm, Doppler color bar range: −16.3 mm/s to 16.3 mm/s).

Consecutively, the application of the handheld OCT imager for *in-vivo* mouse microvascular anastomosis evaluation is tested. To describe how the probe was used in animal experiment, Fig. 5(a) shows the probe held by the surgeon to investigate the experimented mouse vessel. Fig. 5(b) demonstrates how the OCT beam scans across the surgical site with a vessel groove adapted to the vessel. Note that the vessel groove was not implemented in current probe version and will be incorporated into our future version. The slow scanning direction indicated by white arrow repeats every 252 frames to acquire volumetric images of the surgical site. Fig. 6 shows selective frames of simultaneous structure and Doppler imaging of the surgical sites from distal (left) to proximal (right) end of the blood vessel under investigation: Fig. 6(a) (Mouse 1 Artery Cuff, Video S1), Fig. 6(b) (Mouse 1 Vein Cuff, Video S2) and Fig. 6(c) (Mouse 2 Artery Suture, Video S3). Successful blood flow restoration, high vessel lumen patency, minimal lumen narrowing, and thrombosis formation are important parameters for the surgeon to predict successful surgical outcome. To analyze these parameters, all the selected frames were manually segmented and highlighted using ImageJ 1.46r (NIH). The white line encloses the inner lumen of the vessel; the yellow line outlines the blood flowing area within the inner lumen if thrombosis was detected within the lumen. The structure and Doppler images were cropped to a size of 1.5 mm (lateral) by 2 mm (axial). From Doppler images of Fig. 6, we can see clearly that all the blood flow was restored successfully. Strong pulsatile artery flow was clearly visualized. As vessel lumen diameter narrows, flow turbulences were also captured at suture-rich sites. Blood flowing area was segmented using Doppler images. For Fig. 6(a), the area of the artery inner lumen goes from 0.21 mm^2^ (left) to 0.16 mm^2^ (middle) and 0.17 mm^2^ (right). Using the left figure as naive reference–as there is no surgical operation such as cuff and suture–the inner lumen narrowing can by characterized by 23% decrease at middle image and 16% decrease at right image. One probable reason for this inner lumen area drop is that as shown in Fig. 1(b) the top vessel end needs to be expanded and then attached to the bottom vessel end, which will cause the inner lumen area increase for the top vessel. The lumen patency was 100% at reference (left) and drops to 56% at the middle image, and to 80% at the right image, which corresponds to 44% and 20% thrombosis formation crossectionally, respectively. The same analysis was applied to Fig. 6(b). Cuff-based vein inner lumen area are 0.46 mm^2^ (left), 0.09 mm^2^ (middle) and 0.11 mm^2^ (right). The inner lumen narrowing are 80% at the middle and 76% at the right image. This is a relatively large shrinkage in the vein. This might be an indication for changing the cuff with a more appropriate diameter. The patency of the left is 55% while 100% for the middle and right images. For suture-based artery anastomosis, the inner lumen area is 0.18 mm^2^ (left), 0.11 mm^2^ (middle) and 0.13 mm^2^ (right), respectively. The inner lumen narrowing values are 39% at the middle and 28% at the right. The patency was all 100% over the images. No thrombosis was detected. Currently, there is no detailed study of correlating inner lumen area, restenosis, and thrombosis formation percentage with long-term surgical outcome; however, we believe that all these parameters are not only of vital importance for immediate surgical outcome evaluation but also for long-term anastomosis patency prediction. With detailed quantitative image analysis, handheld OCT intraoperative imaging might also help the surgeons to optimize their technique.

Compared to our previous imaging results using 850 nm band OCT systems, 1.3 µm central wavelength shows larger image penetration depth, which enables us to evaluate the inner lumen structure of vessels more accurately. At current study, the vessel inner diameter is around 0.5 mm. For larger size vessels (0.5–1 mm), when penetration depth becomes an issue for imaging, an angular compounding method will be used and evaluated. Currently detailed quantitative blood flow information analysis is limited by the phase wrapping of blood flow at high speed combined with turbulence. Proper correction methods need to be implemented in the future to resolve this issue. Noticeably from the video images there is hand tremor imposed on vessel imaging. A special vessel adapter, such as V-groove on the probe tip will be fabricated so that the probe can rest on the mouse limb without pressing the vessel. Furthermore, three-dimensional motion-compensation algorithms will be developed and applied to remove motion artifacts. Thus, more comprehensive volumetric information of the vessel under investigation can be extracted.

## Conclusion

We have demonstrated a compact user-friendly handheld MEMS scanning mirror-based imager for intraoperative evaluation of microvascular anastomosis and the proof was tested using a mouse model. It can provide real-time simultaneous structure and Doppler flow imaging at a speed of 36 frames per second. The imager performance was carefully characterized experimentally and later evaluated through *in-vivo* mouse femoral vessel anastomosis via both suture and non-suture cuff techniques. Parameters such as vessel inner lumen area, lumen narrowing, and partial thrombosis formation were extracted quantitatively from the imaging results. In conclusion, we believe that the intraoperative application of this handheld OCT imager will greatly improve the quality of microvascular repair and vascular patency and thereby significantly increase long term results after microsurgical vascular repair. Additionally this application allows for real-time performance evaluation and thus provides an optimal platform for in situ performance evaluations in microsurgical trainees.

## Supporting Information

Checklist S1 **ARRIVE checklist.**
(DOCX)

Video S1 **Crossectional inspection of Mouse 1 Artery (using cuff technique) over the surgical site.**
(MP4)

Video S2 **Crossectional inspection of Mouse 1 Vein (using cuff technique) over the surgical site.**
(MP4)

Video S3 **Crossectional inspection of Mouse 2 Artery (using suture technique) over the surgical site.**
(MP4)
